# Epigenetic Reprogramming Mediated by Maternal Diet Rich in Omega-3 Fatty Acids Protects From Breast Cancer Development in F1 Offspring

**DOI:** 10.3389/fcell.2021.682593

**Published:** 2021-06-10

**Authors:** Ata Abbas, Theodore Witte, William L. Patterson, Johannes F. Fahrmann, Kai Guo, Junguk Hur, W. Elaine Hardman, Philippe T. Georgel

**Affiliations:** ^1^Department of Biological Sciences, Marshall University, Huntington, WV, United States; ^2^Cell Differentiation and Development Center, Marshall University, Huntington, WV, United States; ^3^Department of Biochemistry and Microbiology, Marshall University School of Medicine, Huntington, WV, United States; ^4^Department of Biomedical Sciences, University of North Dakota School of Medicine and Health Sciences, Grand Forks, ND, United States

**Keywords:** epigenetic changes, histone post-translational modification (PTM), maternal diet, breast cancer prevention, omega-3 fatty acids

## Abstract

Diets rich in omega-3 fatty acids (FA) have been associated with lowered risks of developing certain types of cancers. We earlier reported that in transgenic mice prone to develop breast cancer (BCa), a diet supplemented with canola oil, rich in omega-3-rich FA (as opposed to an omega-6-rich diet containing corn oil), reduced the risk of developing BCa, and also significantly reduced the incidence of BCa in F1 offspring. To investigate the underlying mechanisms of the cancer protective effect of canola oil in the F1 generation, we designed and performed the present study with the same diets using BALB/c mice to remove any possible effect of the transgene. First, we observed epigenetic changes at the genome-wide scale in F1 offspring of mothers fed diets containing omega-3 FAs, including a significant increase in acetylation of H3K18 histone mark and a decrease in H3K4me2 mark on nucleosomes around transcription start sites. These epigenetic modifications contribute to differential gene expressions associated with various pathways and molecular mechanisms involved in preventing cancer development, including p53 pathway, G2M checkpoint, DNA repair, inflammatory response, and apoptosis. When offspring mice were exposed to 7,12-Dimethylbenz(a)anthracene (DMBA), the group of mice exposed to a canola oil (with omega 3 FAs)-rich maternal diet showed delayed mortality, increased survival, reduced lateral tumor growth, and smaller tumor size. Remarkably, various genes, including BRCA genes, appear to be epigenetically re-programmed to poise genes to be ready for a rapid transcriptional activation due to the canola oil-rich maternal diet. This ability to respond rapidly due to epigenetic potentiation appeared to contribute to and promote protection against breast cancer after carcinogen exposure.

## Introduction

Alpha-linolenic acid (ALA), eicosapentaenoic acid (EPA), and docosahexaenoic acid (DHA) are the three important omega-3 (ω-3) fatty acids (FA) (Lunn and Theobald, 2006; Glick and Fischer, 2013). ALA is an essential FA mainly found in plant oils such as canola oils, flaxseed, and soybean. Linoleic acid (LA) is an omega-6 (ω-6) FA and is also an essential FA that our body could not synthesize. The primary dietary sources of ω-6 FA are vegetable oils, including corn oil, sunflower oil, and soybean oil. Both ω-3 and ω-6 FA are essential structural components of cell membranes and are precursors for various important biochemical conversions. Considering both the benefits and adverse effects of ω-6 FA, a balance between ω-6 and ω-3 FA (optimal ω-6/ω-3 ratio between 1:1 to 1:3) is critical for a healthy lifestyle (Simopoulos, 2009; Gomez Candela et al., 2011; DiNicolantonio and O’Keefe, 2018). Numerous studies provide preclinical evidence linking the ω-3 to ω-6 FA ratio in the diet with cancer development (Pizato et al., 2005; Hardman, 2007; Kang and Liu, 2013; Abel et al., 2014; Diorio and Dumas, 2014; Zanoaga et al., 2018).

Over the last 15 years, an increasing number of publications have linked various dietary compounds to the long-term protection of individuals against multiple types of cancers (Syed et al., 2008; Turati et al., 2015; Vanamala, 2017; Koh et al., 2020; Saini et al., 2020; Steck and Murphy, 2020). Notably, both maternal and paternal diets contribute, in a trans-generational manner, to this observed protective effect (Ion et al., 2010; Fleming et al., 2018; Li et al., 2018). Among the dietary compounds investigated, ω-3 and ω-6 FA have received significant interest, as using oil high in ω-3 and low in ω-6 FA may provide an easy and affordable way to reduce the incidence of several types of cancer, including breast cancer (BCa) (Karmali et al., 1993; Huang et al., 1996; Hardman et al., 1999; Hardman, 2004), possibly through ω-3 FA’s anti-inflammatory properties (Patterson and Georgel, 2014). More recently, as nutrients are unlikely to trigger mutations, researchers have shifted their interest to a potential epigenetic mode of action to explain the diet-mediated gene expression changes (Choi and Friso, 2010; Abbas et al., 2013, 2016; Bishop and Ferguson, 2015; Remely et al., 2015).

It is evident that ω-3 FA can reduce the risk of developing various complex diseases, including cancer (Hardman, 2014; Witte and Hardman, 2015; Simopoulos, 2016). An earlier report from our group using BCa transgenic C3(1)-TAg mice indicated that a canola oil supplemented maternal diet (ω-3 FA rich) significantly reduced the incidence of BCa in F1 offspring (Ion et al., 2010). Here, we sought to comprehensively investigate the effect of a canola oil-rich maternal diet compared to that of a corn oil-rich maternal diet and the underlying protective mechanisms preventing BCa development after DMBA treatment using non-transgenic BALB/c mice. The use of normal BALB/c mice allowed us to remove any possible effects of the transgene or of a developing mammary gland tumor from the genetic profile of the mice.

## Results

### Maternal Diet Induces Genome-Wide Epigenetic Changes in the Mammary Tissue of F1 Generation Mice

**FIGURE 1:**
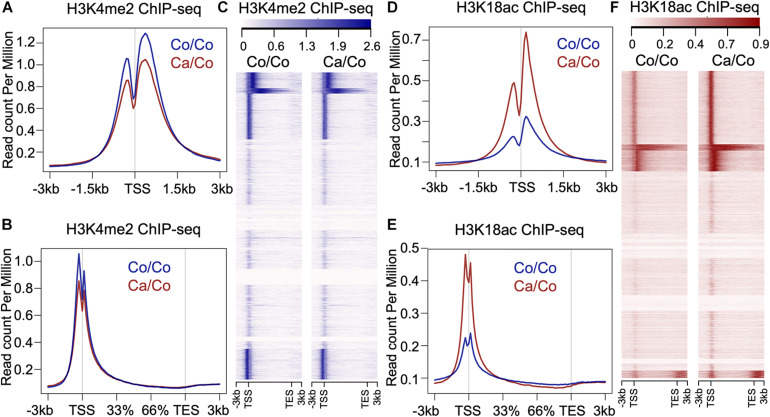
Effects of maternal diets on histone post-translational modifications (PTMs) in offspring breast tissue. **(A)** Metagene plot showing histone H3K4me2 ChIP-seq signals around transcription start site (TSS, ±3 kb) in the breast tissue of F1 generation mice whose mothers were fed either corn (Co/Co) or canola (Ca/Co) oil-rich diets. **(B)** Metagene plot and **(C)** heatmap showing histone H3K4me2 ChIP-seq signals at gene bodies (–3 kb from TSS to +3 kb beyond TES, transcription end site) in the breast tissue of F1 generation mice whose mothers were fed either corn (Co/Co) or canola (Ca/Co) oil-rich diets. **(D)** Metagene plot showing histone H3K18ac signals around TSS (±3 kb) in the breast tissue of F1 generation mice whose mothers were fed either corn (Co/Co) or canola (Ca/Co) oil-rich diets. **(E)** Metagene plot and **(F)** heatmap showing histone H3K18ac ChIP-seq signals at gene bodies in the breast tissue of F1 generation mice whose mothers were fed either corn (Co/Co) or canola (Ca/Co) oil-rich diets.

To understand if maternal diets enriched with either canola oil or corn oil can modulate histone post-translational modification (PTM) in the F1 generation, we first screened the global changes in histone PTMs known to affect gene expression, such as H3K9me2, H3K9me3, H3K18ac, and H3K4me2, as well as H4K5ac, H4K8ac, H4K12ac, H4K16ac, and panH4ac using western blot (Supplementary Figure 1). We observed a statistically significant increase in acetylation of histone 3 at lysine 18 in mammary tissue of the F1 mice from mothers fed a canola oil-rich diet (Ca/Co) compared to mothers fed a corn oil-rich diet (Co/Co) (*P*-value = 0.005) along with an increase in H4K12ac and H4K16ac marks (Supplementary Figure 1). Since the increase in global H3K18ac was statistically significant, and its association with gene transcription is well-known, we decided to perform chromatin immunoprecipitation-sequencing (ChIP-seq) to elucidate genome-wide changes. We also noted an increase in H3K4me2, and though it was not statistically significant, we included it in our ChIP-seq experiments due to its role in gene expression and regulation. We performed H3K4me2 and H3K18ac ChIP-seq using mammary gland tissue samples of F1 mice whose mothers were fed either corn (Co/Co) or the canola (Ca/Co) oil-rich diets (Figure 1 and Supplementary Figure 2). We observed a slight decrease in the H3K4Me2 signal around the transcription start site (TSS) in our Ca/Co mice compared to Co/Co mice (Figures 1A–C). Remarkably, the H3K18ac signal was enriched around TSS in F1 mice from Ca/Co group compare to the Co/Co mice (Figures 1D–F). Next, we analyzed H3K4me2 and H3K18ac ChIP-seq for lincRNA genes, microRNA genes, and enhancers and compared them between Ca/Co and Co/Co mice. We observed a notable increase in H3K18ac histone marks around TSS of lincRNA genes with a slight decrease in H3K4me2 level (Supplementary Figure 3A). Surprisingly, H3K18ac histone PTM was decreased at enhancer regions, but no noticeable change in H3K4me2 PTM level over enhancer regions was observed (Supplementary Figure 3B). We did not observe any noteworthy changes in micro RNA (miRNA) genes (Supplementary Figure 3C). These observations, altogether, suggest that the maternal ω-3 FA rich diet can significantly impact the genome-wide epigenetic landscape changes in offspring and potentially modulate gene expression patterns in F1 mice.

### Maternal Diets Modulate Global Gene Expression Patterns in F1 Offspring

**FIGURE 2:**
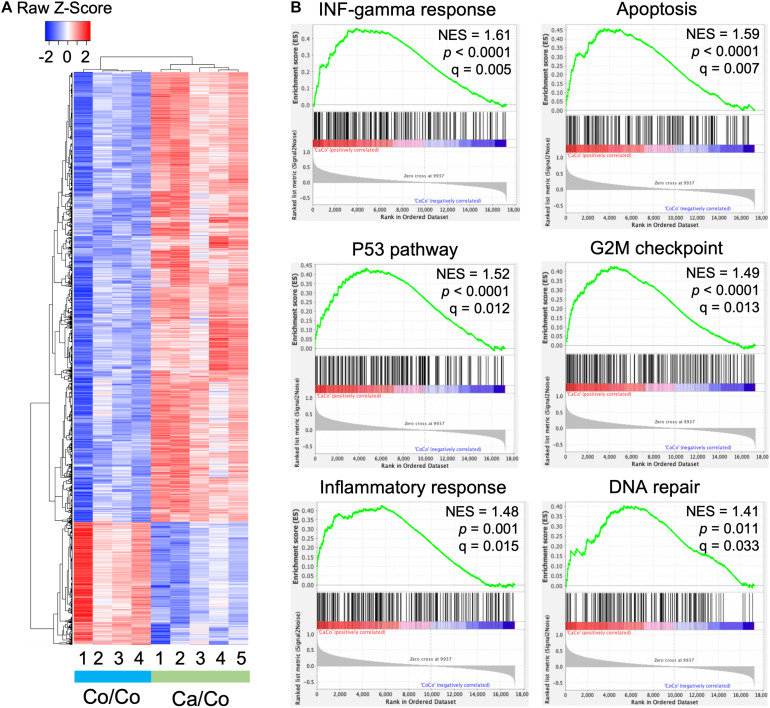
Maternal diets modulate genome-wide gene expression patterns in offspring. **(A)** Heatmap showing differential gene expression in breast tissue samples in offspring mice whose mothers were fed either corn (Co/Co) or canola (Ca/Co) oil-rich diets by microarray analysis. We observed 2,767 and 759 genes that exhibited twofold or more over-expression or under-expression, respectively (*P*-adj < 0.05). **(B)** GSEA analysis showing enrichment of various pathways due to differential gene expression (Ca/Co vs. Co/Co; NES, normalized enrichment score).

We performed microarray analysis to measure the differentially expressed genes in mammary gland tissues of Ca/Co mice compared to Co/Co mice (Figure 2 and Supplementary Figure 4). Among the observed differentially expressed genes, 2,767 genes were over-expressed (≥2-fold, *P*-adj < 0.05), and 759 genes were under-expressed (≥2-fold, *P*-adj < 0.05) (Figure 2A). Next, we performed Gene Set Enrichment Analysis (GSEA) using Molecular Signatures Database (MSigDB) for our normalized microarray gene expression datasets. Several pathways were significantly upregulated in Ca/Co mice (Supplementary Figure 5) such as IL2-STAT5 signaling pathway involved in mammary gland development and lactogenesis (Liu et al., 1997; Supplementary Figure 5B); however, no significantly downregulated pathways were observed. Remarkably, among the top significantly enriched pathways in Ca/Co mice were interferon-gamma response, apoptosis, p53 pathway, G2M checkpoint, DNA repair, and inflammatory response (Figure 2B). Activation of these pathways in Ca/Co mice compared to Co/Co mice suggest better homeostatic controls that suppress aberrant cell proliferation in tumorigenesis. These observations signify anti-cancer properties of omega-3 FA and further provide the first line of evidence for transcriptomic changes favoring the blockade of neoplastic cells in F1 offspring.

### Maternal ω-3 FA Diet Induces Epigenetic Changes in Nucleosomes Around TSS of Differentially Expressed Genes

**FIGURE 3:**
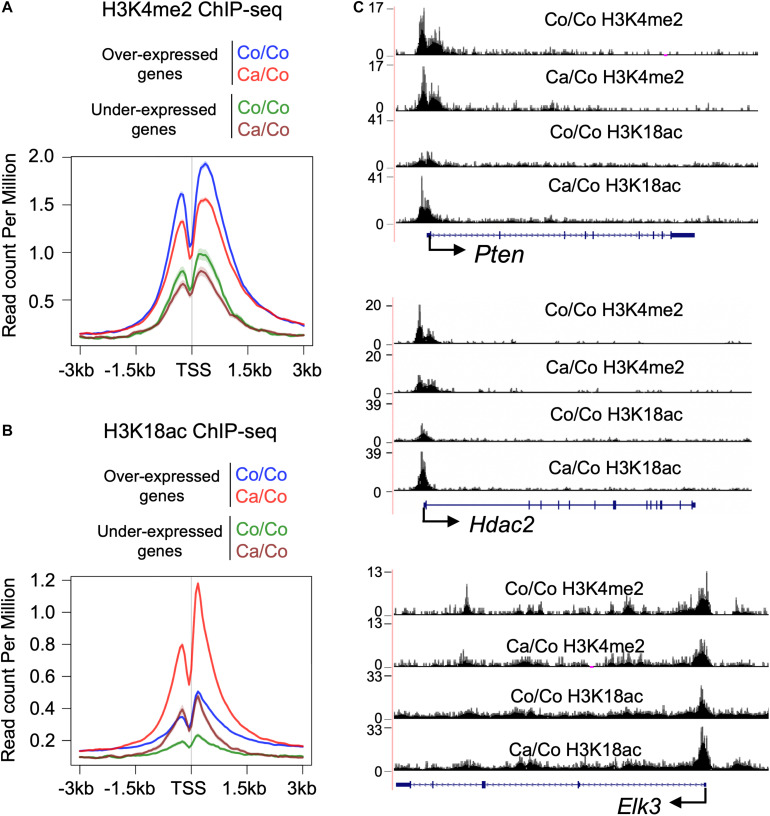
Histone PTMs at TSS of the differentially expressed genes. Metagene plot showing histone H3K4me2 **(A)** and H3K18ac **(B)** ChIP-seq signals at TSS (±3 kb) of differentially expressed genes in the breast tissue of F1 generation mice whose mothers were fed either corn (Co/Co) or canola (Ca/Co) oil-rich diets. **(C)** UCSC genome browser tracks representing the changes in H3K4me2 and H3K18ac signals around TSS due to maternal diets are shown for *Pten* (over-expressed), *Hdac2* (unchanged), and *Elk3* (under-expressed) genes.

Epigenetic changes, especially specific subsets of histone PTMs have been shown to be associated with genes’ transcriptional status (Jenuwein, 2001). We, therefore, analyzed H3K4me2 and H3K18ac ChIP-seq signals at TSS of differentially expressed genes to better understand the impact of maternal diets induced epigenetic changes on gene transcription in F1 mice mammary tissue. As expected, overall H3K4me2 histone marks around TSS were higher in over-expressed genes compare to under-expressed genes in both Ca/Co and Co/Co mice (Figure 3A). However, enrichment of H3K4me2 marks was slightly decreased in both over-expressed and under-expressed genes around TSS in mammary tissue of Ca/Co mice compare to Co/Co mice (Figure 3A). Next, we looked at H3K18ac ChIP-seq signals around TSS of differentially expressed genes. We observed a large noticeable increase in acetylation of histone H3K18 around TSS in over-expressed genes in mammary tissue of F1 generation of Ca/Co mice compare to Co/Co mice (Figure 3B). Note that the increase that we observed was often at TSS which already displayed H3K18ac signal. The Ca/Co seemed to accentuate the signal. There was a slight increase in H3K18ac ChIP-seq signals around TSS of under-expressed genes as well. An examination of representative genes, *Pten* (over-expressed), *Hdac2* (unchanged), and *Elk3* (under-expressed) using the UCSC genome browser further corroborates our observations of maternal diet-induced alterations of histone marks around TSS (Figure 3C). To understand if there were any specific patterns or correlations of non-differentially expressed genes (Ca/Co vs. Co/Co) with H3K4me2 and H3K18ac histones marks around TSS, we calculated H3K4me2 and H3K18ac counts around TSS (±500 bp) to plot cumulative frequency and scattered plots (Supplementary Figure 6). Although there was a subset of genes that was differentially expressed and showed alterations in histone marks (Figure 3 and Supplementary Figure 6), no specific patterns were observed in the histone modulations of the remaining genes that exhibited no change in expression level (Supplementary Figures 6A,B). Since the increase in H3K18ac level is not absolutely correlated with enhanced expression (only a subset of genes was over-expressed), this raises the possibility that the canola oil-rich maternal diet induces epigenetic potentiation in these genes in a context-dependent manner, possibly contributing to maintenance of cellular homeostasis.

### Maternal Diets Modulate the Carcinogenic Effects of DMBA on Offspring Mammary Glands

**FIGURE 4:**
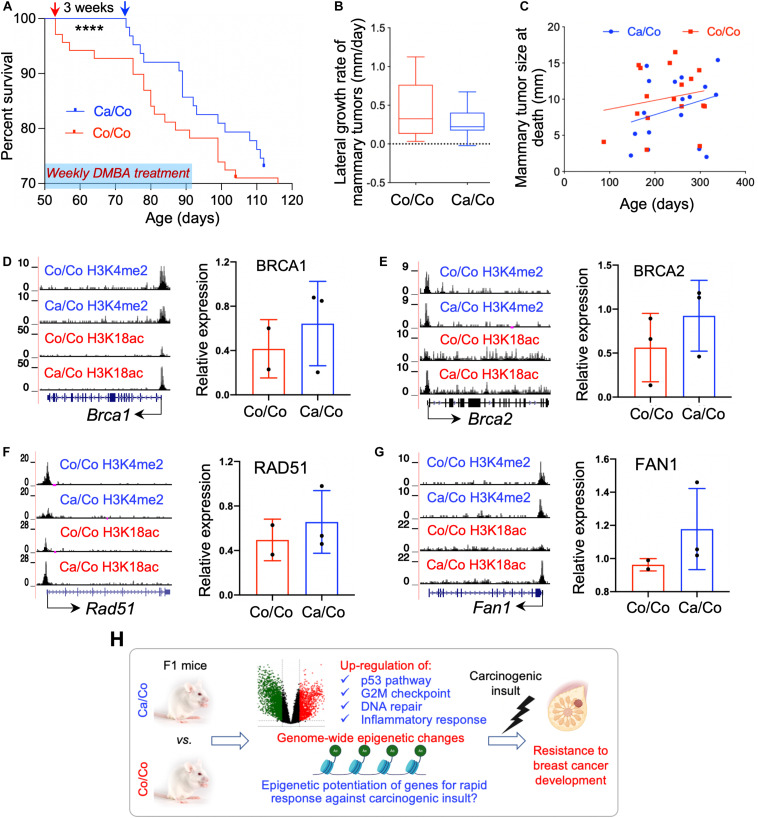
Maternal diets modulate the effects of DMBA on offspring mammary tissue. **(A)** Survival curve showing 3 weeks delay in mortality (***P-value < 0.0001) after DMBA treatment in F1 generation mice whose mothers were fed canola (Ca/Co) oil in comparison to corn (Co/Co) oil-rich diets, however, overall survival is not statistically significant. Red and blue arrows showing the time of first mortality in Co/Co and Ca/Co mice respectively after DMBA treatment. **(B)** Lateral growth rate of mammary tumors (mm/day) after DMBA treatment in F1 mice (Tukey; *P*-value, ns). **(C)** Mammary tumor size at the time of death (*P*-value, ns). **(D)** UCSC genome browser tracks showing maternal diets induced epigenetic changes (potentiation) in the *Brca1* gene (left, no DMBA) and BRCA1 protein expression (right, quantified using ImageJ) after DMBA treatment (*t*-test; *P*-value, ns). **(E)** UCSC genome browser tracks showing maternal diets induced epigenetic changes (potentiation) in the *Brca2* gene (left, no DMBA) and BRCA2 protein expression (right, quantified using ImageJ) after DMBA treatment (*t*-test; *P*-value, ns). **(F)** UCSC genome browser tracks showing maternal diets induced epigenetic changes (potentiation) in the *Rad51* gene (left, no DMBA) and RAD51 protein expression (right, quantified using ImageJ) after DMBA treatment (*t*-test; *P*-value, ns). **(G)** UCSC genome browser tracks showing maternal diets induced epigenetic changes (potentiation) in the *Fan1* gene (left, no DMBA) and FAN1 protein expression (right, quantified using ImageJ) after DMBA treatment (*t*-test; *P*-value, ns). **(H)** Carton describing differential gene expression and epigenetic potentiation providing resistance to BCa development in F1 generation mice whose mothers were fed canola rich diet (Ca/Co). ns, not significant.

To test the effect of maternal diets (canola vs. corn oil-rich diets) on the protection against DMBA induced carcinogenesis, F1 mice were treated with DMBA (weekly dose of 1 mg for 6 weeks). Surprisingly, we observed a 3-week delay in DMBA induced mortality in F1 generation mice whose mothers were fed canola oil (Ca/Co) in comparison to corn (Co/Co) oil-rich diets (Figure 4A). This early delay in mortality in Ca/Co group was statistically significant (*P*-value < 0.0001), however, the overall survivals were not statistically different. Eventually, all of the mice developed tumors and became moribund; still, tumor development in Co/Co mice was faster than in Ca/Co mice (Supplementary Figure 7A). The lateral growth rate of mammary tumors was slower (Figure 4B) and size of mammary tumors was relatively less in Ca/Co mice compared to Co/Co mice, albeit not statistically significant (Figure 4C). Since maternal diets epigenetically modulated various gene promoters, we performed protein expression analysis using western blotting to determine if the expression of proteins responsible for DNA damage repair and epigenetic remodeling were altered after carcinogen exposure. We found that increased H3K18ac levels around TSS were correlated with slightly increased levels of BRCA1, BRCA2, RAD51, and FAN1 proteins in the Ca/Co mice compared to the Co/Co mice in response to DMBA; however, changes in protein levels were not statistically significant (Figures 4D–G and Supplementary Figure 7B). Notably, the transcriptional expression of *Brca1* was decreased (*P*-value < 0.01), and *Fan1* was unchanged in the Ca/Co mice compared to Co/Co mice before DMBA treatment (basal level in F1, Supplementary Figure 8). Furthermore, protein expression of PRMT2 was relatively increased (*P*-value, ns) but CHD1 and SMARCA5 was unchanged in Ca/Co compared to Co/Co mice (Supplementary Figure 7C); however, transcriptional levels of *Prmt2* and *Chd1* genes were unchanged and *Smarca5* was significantly increased (*P*-value < 0.01) in the Ca/Co mice before DMBA treatment (Supplementary Figure 8). Altogether, these findings suggest that the canola oil-rich maternal diets epigenetically modulate transcriptional expression and epigenetically potentiate various genes, possibly for rapid activation to protect from mammary tumor development in the event of carcinogenic insult.

## Discussion

Epigenetic inheritance in plants, nematodes, and *Drosophila* has already been demonstrated, but how environmental cues transmit to offspring in mammals, if they do, is controversial (Heard and Martienssen, 2014; Boskovic and Rando, 2018; Horsthemke, 2018). However, it is apparent that the environment, including diets, certainly affects gene expression patterns and influences physiopathology. Recently, Xavier et al. (2019) reviewed the impact of various environmental factors, including diets on epigenetic states, and how they can be hereditably transmitted. More precisely, recent evidence has shown that parental diets can influence the outcome of BCa through transgenerational epigenetic inheritance (da Cruz et al., 2020). The present study evaluated the influence of maternal diet on BCa development in F1 offspring using BALB/c mice and investigated the associated genome-wide epigenetic and transcriptional changes.

There is a large body of evidence suggesting the chemo-preventive effect of ω-3 FA against BCa and other cancer development [see recent reviews and meta-analysis (Mokbel and Mokbel, 2019; Nindrea et al., 2019a,b; Donovan et al., 2020; Yurko-Mauro et al., 2020)]. Hardman’s group earlier reported that maternal diet supplemented with canola oil (ω-3 FA-rich) reduced tumor incidence and growth in C3(1)-TAg mice offspring (Ion et al., 2010), suggesting the involvement of possible transgenerational epigenetic mechanisms. Our data, showing the effect of maternal diet on genome-wide histone modifications (Figure 1) in F1 BALB/c mice, provides first-hand evidence corroborating the above findings. Remarkably, the transcriptomic changes involve the differential expression of ∼3,500 genes, enriched in multiple molecular pathways associated with preventing/suppressing cancer incidence/growth (Figure 2 and Supplementary Figure 5). The unpredicted large number of genes affected suggest a nearly genome-wide impact of ω-3 FA-rich maternal diet. Consistent with our results, it has been shown that a high ω-3 FA-rich diet can lead to the inhibition of the enzyme acetyl-CoA carboxylase (Willumsen et al., 1993), in turn leading to an increased pool of acetyl-CoA, a acetyl donor for histone acetylation. An increased acetyl-CoA level due to attenuation of acetyl-CoA carboxylase enzyme can lead to the global increase in histone acetylation (Galdieri and Vancura, 2012). This change in global histone acetylation is congruent with the observed increase in H3K18ac that we reported in this study, But, importantly, our results suggest that the change in global histone acetylation that is of maternal origin appears to be passed onto the F1 mice of our Ca/Co group.

Histone modifications around TSS play a crucial role in gene transcription. An open chromatin state (euchromatin) around the TSS is created or maintained for the recruitment of the general transcriptional machinery, as well as various gene-specific transcriptional factors necessary to regulate transcriptional homeostasis. A large increase of H3K18ac with a decrease in H3K4me2 marks around TSS of over-expressed genes (Figure 3, ∼80% of total differentially expressed genes are over-expressed) indicates that epigenetic regulation is a primary driver of the effects of ω-3 FA rich maternal diets. These results are consistent with recent studies that demonstrate the epigenetic modifying potential of ω-3 FA (Tremblay et al., 2017; Amatruda et al., 2019; Gonzalez-Becerra et al., 2019; Ceccarelli et al., 2020); however, to the best of our knowledge, this is the first study comprehensively showing the global impact of ω-3 FA-rich maternal diets on F1 offspring’s epigenome and transcriptome.

The ω-3 FA-rich maternal diets in addition to modulating F1 offspring’s epigenome, it also incredibly provides protection again early carcinogenic insults (Figure 4 and Supplementary Figure 7). As our data indicated, epigenetic changes, more specifically the increase in H3K18ac at TSS were located over a subset of important DNA damage response genes. The activation of such genes is likely to be associated with an early response against DMBA induced carcinogenesis (Figures 4D–G). These results suggest that the ω-3 FA-rich maternal diet causes epigenetic potentiation of genes in F1 offspring, setting them ready to rapidly express DNA repair and cell cycle controlling genes after a carcinogenic insult.

In conclusion, our study provides an in-depth analysis of mechanisms of action involving genome-wide epigenetic changes mediated by the ω-3 FA-rich maternal diet onto F1 offspring. These changes in both expression and epigenetics potentiation of genes important in cancer-fighting pathways contribute to providing resistance to BCa development (Figure 4H). However, future studies are needed to further pin-point the diet-induced specific transgenerational epigenetic effects, and also extend the results to F2 and F3 generations, and their role in preventing various chronic illnesses, including cancers.

## Materials and Methods

### Animal Experiments

BALB/c mice (around 4 weeks old) were purchased from the Jackson Laboratory (Bar Harbor, ME, United States). The animal protocol describing the mouse treatment and use was reviewed and approved by the Marshall University Institutional Animal Care and Use Committee (IACUC). Mice were used in compliance with the National Institutes of Health (NIH) recommendations published in the “Guide for the Care and Use of Laboratory Animals”^1^. Mice were randomized and 15 female mice were placed on AIN-93 diet containing 10% corn oil, 15 female mice were placed on a diet containing 10% canola oil. After 2 weeks, mice were bred with male BALB/c mice to produce 200 experimental female mice (100 from corn oil and 100 from canola oil supplemented maternal diet group). At weaning, all pups were placed on the corn oil diet, thus the only time any pups were exposed to the canola oil diet was during gestation and lactation. At 50 days of age, the female pups were randomized, half of each maternal diet group were treated with 7,12-Dimethylbenz(a)anthracene (DMBA). Mammary gland tumors were induced using DMBA, beginning at 50 days of age. DMBA (10 mg/ml in sesame seed oil) treated pups (*n* = 50) received 0.1 ml of DMBA by gavage once per week for 6 weeks. Control pups (*n* = 50) without DMBA received only 0.1 ml of vehicle (sesame seed oil). After 6 weeks, non-DMBA control pups provided mammary tissue for transcriptomic (microarray) and epigenetic (ChIP-seq) studies to examine genome-wide differences that are the result of the maternal diet and that are not influenced by the carcinogen nor by a tumor. DMBA-treated pups were palpated weekly until a tumor was detected. We followed DMBA-treated pups for the time of the first detection of tumor incidence and multiplicity. After detection, all tumors were measured three times weekly to develop tumor growth curves.

### Microarray for Gene Expression

Total RNA was extracted from cells using the RNeasy kit (Qiagen, Germantown, MD, United States). On-column DNase treatment was given following the procedure provided by the manufacturer. RNA quality was assessed via the Agilent 2100 Bioanalyzer (Agilent Technologies, Santa Clara, CA, United States). Microarray analysis was performed using Agilent Gene Expression Microarray Platform (SurePrint G3 Mouse GE 8 × 60K Microarray) at Marshall University Genomics and Bioinformatics Core following the manufacturer recommendations. Processing of the microarray, including normalization and differential expression analysis, was performed using GeneSpring GX (Agilent Techonolgies, United States). Principal component analysis (PCA) was performed to examine the overall similarity among the samples at the gene expression level. *t*-test was used to identify differential gene expression between the Ca/Co and Co/Co groups with Benjamini–Hochberg multiple testing corrected *P*-value < 0.05 as the significant cut-off. Differentially expressed genes were further curated by removing “unknown” genes, cDNA clones, etc. GSEA (Subramanian et al., 2005) was performed to identify over-represented biological functions in the differential gene expression in terms of the signatures in MSigDB (Liberzon et al., 2011).

### ChIP-Sequencing and Analysis

Chromatin immunoprecipitation-sequencing was performed on pooled samples. Briefly, mammary tissues were dissected into small pieces and crosslinked using formaldehyde (1% w/v, methanol-free) followed by homogenization and chromatin preparation using a Covaris sonicator (Covaris, Woburn, MA, United States). Chromatin was immunoprecipitated using an appropriate antibody, washed, reverse-crosslinked, and DNA isolated using the phenol-chloroform method. Precipitated DNA was QC’ed by quantitative PCR using Mouse ChIP Control qPCR Primer Sets (Active Motiff, Carlsbad, CA United States). For ChIP-seq, 10 ng precipitated DNA was used to prepare the library using Illumina TruSeq ChIP library preparation kit following the manufacturer recommendations. Libraries were quantified for cluster generation using KAPA Library Quantification Kit (Kapa Biosystems, Wilmington, MA, United States). Sequencing was performed using Illumina HiSeq2500 using 50-bp single-end rapid run format. Sequencing quality was initially checked by running FastQC. Sequencing reads were aligned against mm9 by using Bowtie2. BAM files were normalized by depth after removal of PCR duplicates and blacklisted regions. Peaks were called using MACS2 with default parameters. For heatmap and metagene plots, ngs.plot program was used. UCSC genome browser was used to visualize individual gene tracks.

### Western Blot

Protein lysates were prepared from mammary tissues using a standard RIPA lysis buffer containing a protease inhibitor cocktail (Roche). Lysates were electrophoresed using SDS-PAGE, transferred to ECL nitrocellulose membranes (Amersham), and probed using specific antibodies. HRP-conjugated secondary antibodies were used, and our blots were developed using the ECL detection system (Thermo Scientific) combined with ECL hypersensitive films. The densitometry analysis was performed using the Alpha Innotech FluorChem^TM^ IS 9000 software.

### Statistics

Prism^TM^ was used for analyses of data and graph preparation. Time-to-death or to-detection of mammary gland tumors was analyzed by a log-rank (Mantel–Cox) test. These “survival” curves also provided calculation of median times. Prism^TM^ was used to further analyze the survival curve data to generate linear regression analyses of portions of the data. For box plot, box and whiskers graphs were plotted using the Tukey method (two-tailed Mann–Whitney U test was used to calculate *P*-value). The middle line in the box indicates the median, whiskers indicate the highest and lowest values within 1.5 × IQR (inter-quartile distance between the 25th and 75th percentiles) up and down from the box. Bar graphs are plotted using mean with SD, and *t*-tests were used to calculate *P*-values.

## Data Availability Statement

The datasets presented in this study can be found in online repositories. The names of the repository/repositories and accession number(s) can be found below: NCBI GEO; GSE169115.

## Ethics Statement

The animal study was reviewed and approved by the Marshall University School of Medicine Institutional Animal Care and Use Committee.

## Author Contributions

PG and WH conceptualized the study. AA, TW, WP, and JF performed the experiments. AA, KG, and JH performed bioinformatic data analyses. AA and PG drafted the manuscript. All authors critically revised the manuscript and gave final approval.

## Conflict of Interest

The authors declare that the research was conducted in the absence of any commercial or financial relationships that could be construed as a potential conflict of interest.

## Footnotes

1 https://grants.nih.gov/grants/olaw/guide-for-the-care-and-use-of-laboratory-animals.pdf
