# Supplementary material for: Epigenetic Reprogramming Mediated by Maternal Diet Rich in Omega-3 Fatty Acids Protects From Breast Cancer Development in F1 Offspring
Source: Front Cell Dev Biol. 2021 Jun 10;9:682593. doi: 10.3389/fcell.2021.682593 (PMC8222782; doi:10.3389/fcell.2021.682593)
Supplement: Supplementary file 1 [file Data_Sheet_1.PDF]

A

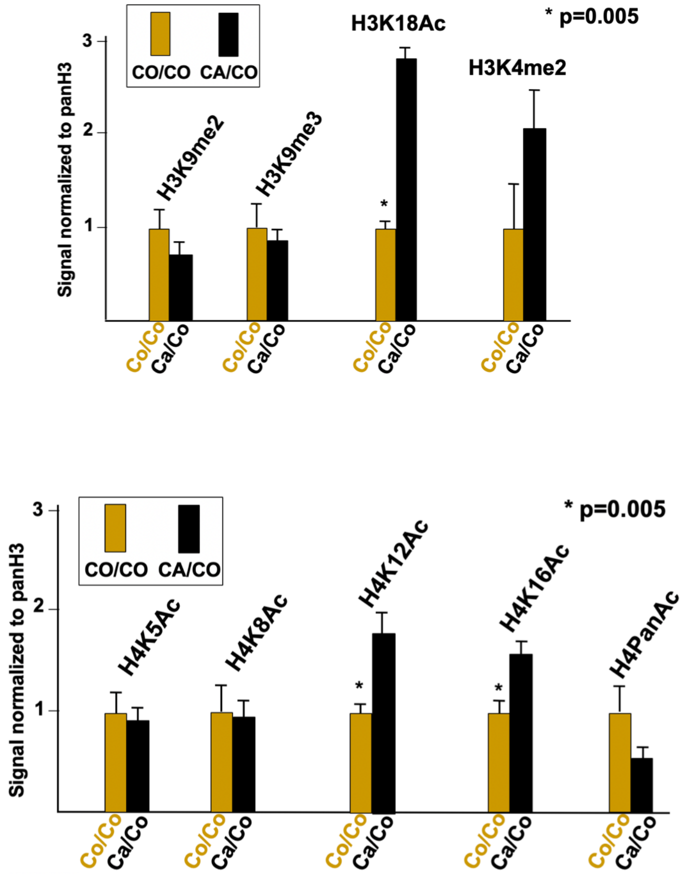

B

Supplementary Fig. 1. Maternal diets affect histone modifications in offspring. Graphs representing changes in various **A)** histone H3, and **B)** histone H4 marks quantified using ImageJ after Western Blotting in breast tissues of F1 generation mice whose mothers were fed either corn (Co/Co) or canola (Ca/Co) oil-rich diets (mean with SD, n=3).

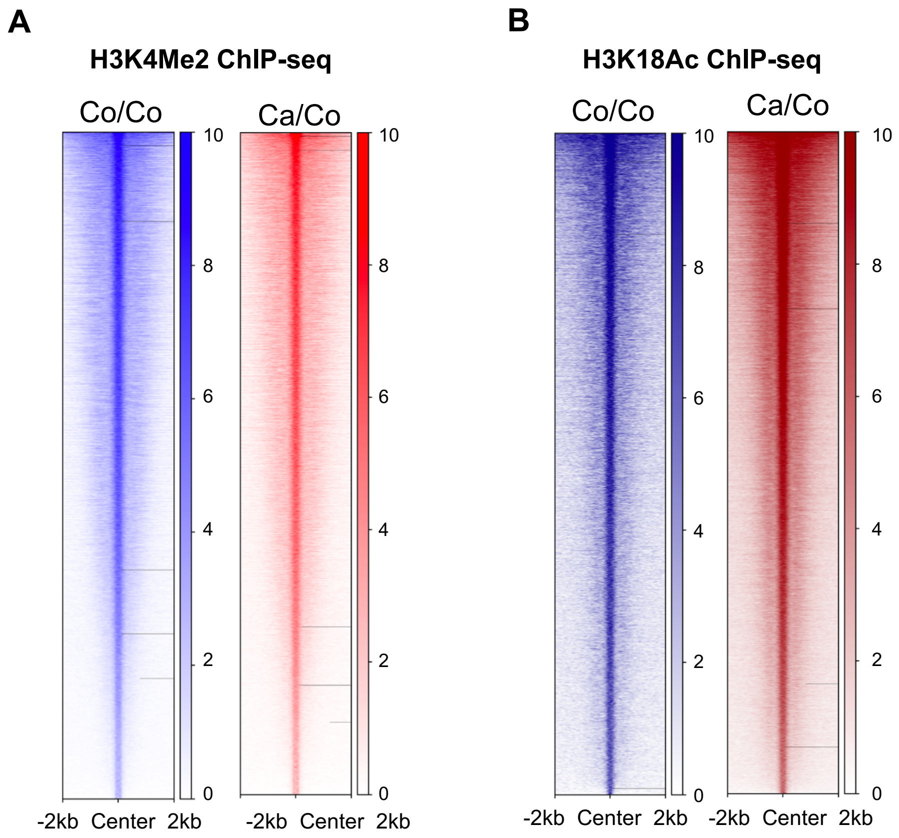

Supplementary Fig. 2. Effects of maternal diets on histone modifications in F1 generation mice. Heatmaps representing MACS2 peaks in **A)** H3K4me2, and **B)** H3K18ac ChIP-seq in breast tissue of F1 generation mice whose mothers were fed either corn (Co/Co) or canola (Ca/Co) oil rich diets.

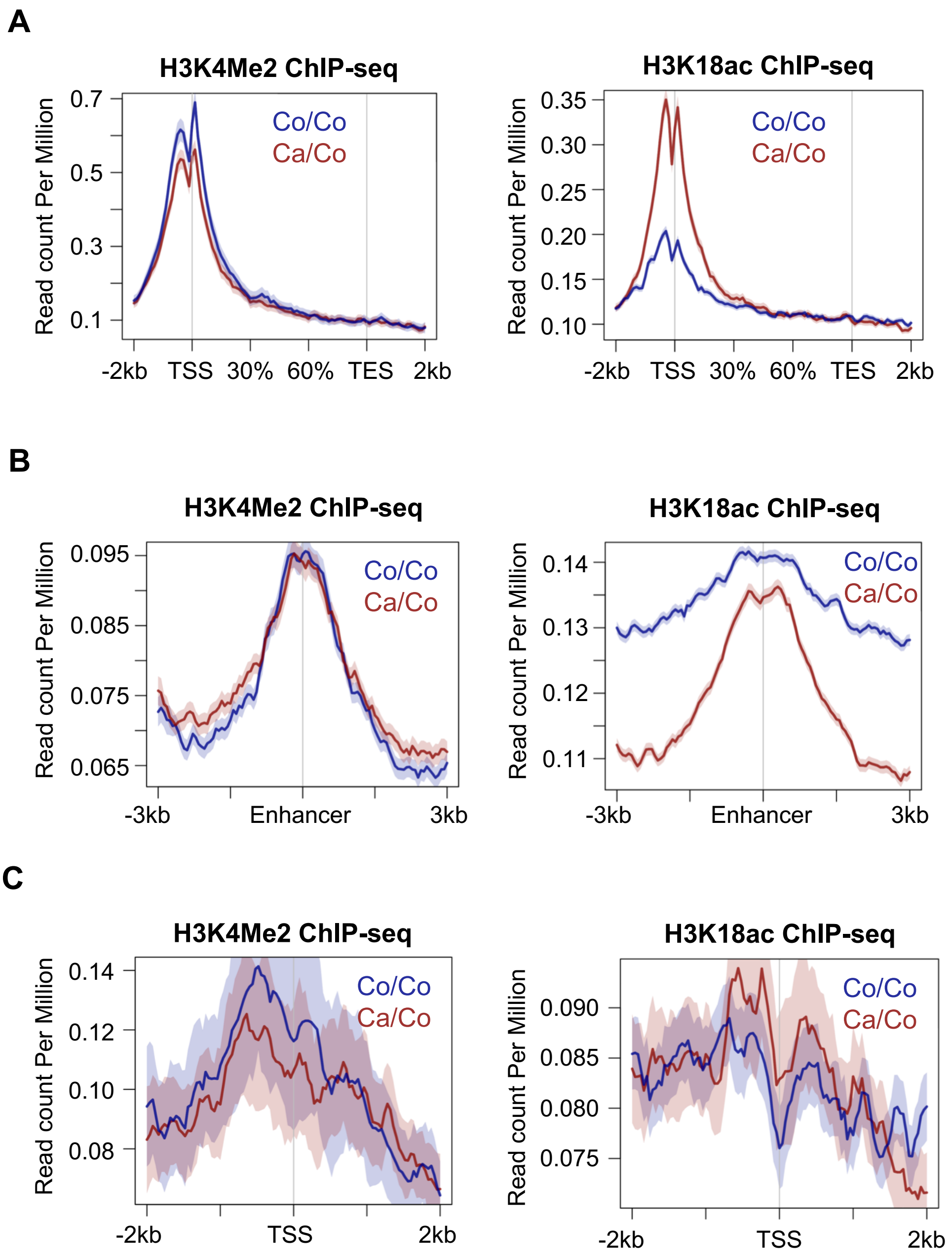

Supplementary Fig. 3. Changes in genome-wide histone modifications in F1 generation due to maternal diets . Metagene plots representing H3K4me2 and H3K18ac ChIP-seq signals **A)** at lincRNA gene-bodies, **B)** around enhancers, and **C)** at miRNA TSS in breast tissue of F1 generation mice whose mothers were fed either corn (Co/Co) or canola (Ca/Co) oil rich diets.

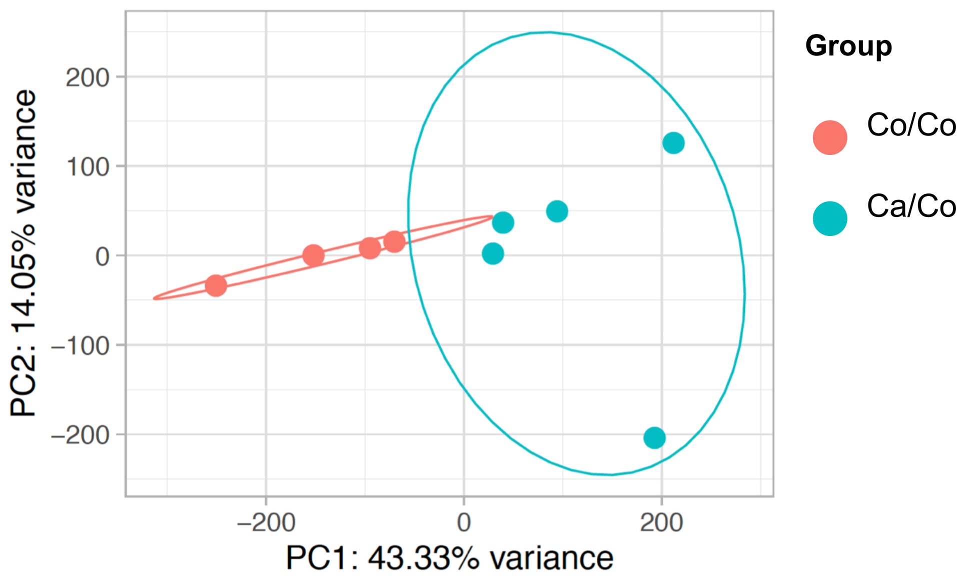

Supplementary Fig. 4. PCA plot representing correlation of replicates form Co/Co (n=4) and Ca/Co (n=5) groups used in Microarray analysis.

A

Gene sets	ES	NES	p-val	q-val
HALLMARK_IL2_STAT5_SIGNALING	0.51	1.82	0	0.002
HALLMARK_ALLOGRAFT_REJECTION	0.51	1.79	0	0.001
HALLMARK_TNFA_SIGNALING_VIA_NFKB	0.48	1.68	0	0.004
HALLMARK_E2F_TARGETS	0.48	1.67	0	0.003
HALLMARK_ESTROGEN_RESPONSE_EARLY	0.46	1.63	0	0.006
HALLMARK_KRAS_SIGNALING_UP	0.46	1.61	0	0.006
HALLMARK_INTERFERON_GAMMA_RESPONSE	0.46	1.61	0	0.006
HALLMARK_APOPTOSIS	0.46	1.59	0	0.007
HALLMARK_MTORC1_SIGNALING	0.45	1.57	0	0.009
HALLMARK_IL6_JAK_STAT3_SIGNALING	0.47	1.56	0.001	0.009
HALLMARK_ANDROGEN_RESPONSE	0.46	1.54	0	0.012
HALLMARK_MYC_TARGETS_V2	0.49	1.53	0.012	0.012
HALLMARK_P53_PATHWAY	0.43	1.52	0	0.012
HALLMARK_ESTROGEN_RESPONSE_LATE	0.43	1.52	0	0.012
HALLMARK_WNT_BETA_CATENIN_SIGNALING	0.51	1.52	0.016	0.011
HALLMARK_G2M_CHECKPOINT	0.43	1.5	0	0.014
HALLMARK_INFLAMMATORY_RESPONSE	0.42	1.48	0.001	0.015
HALLMARK_PROTEIN_SECRETION	0.43	1.44	0.013	0.026
HALLMARK_ANGIOGENESIS	0.48	1.42	0.052	0.031
HALLMARK_TGF_BETA_SIGNALING	0.45	1.41	0.039	0.032
HALLMARK_DNA_REPAIR	0.4	1.41	0.011	0.033
HALLMARK_PI3K_AKT_MTOR_SIGNALING	0.41	1.4	0.017	0.036
HALLMARK_CHOLESTEROL_HOMEOSTASIS	0.42	1.37	0.051	0.046
HALLMARK_NOTCH_SIGNALING	0.48	1.37	0.067	0.045

B

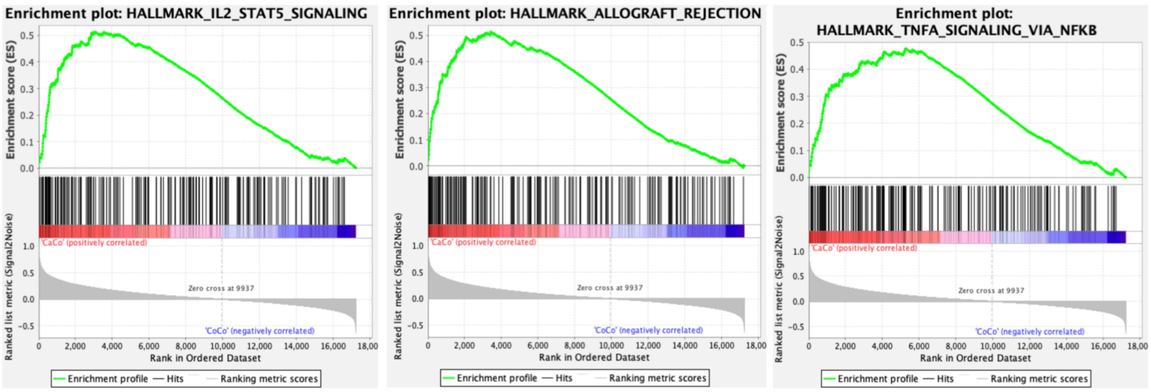

Supplementary Fig 5. A) GSEA analysis of differentially expressed genes (Ca/Co vs Co/Co). **B)** Representative GSEA plot showing enrichment of top pathways.

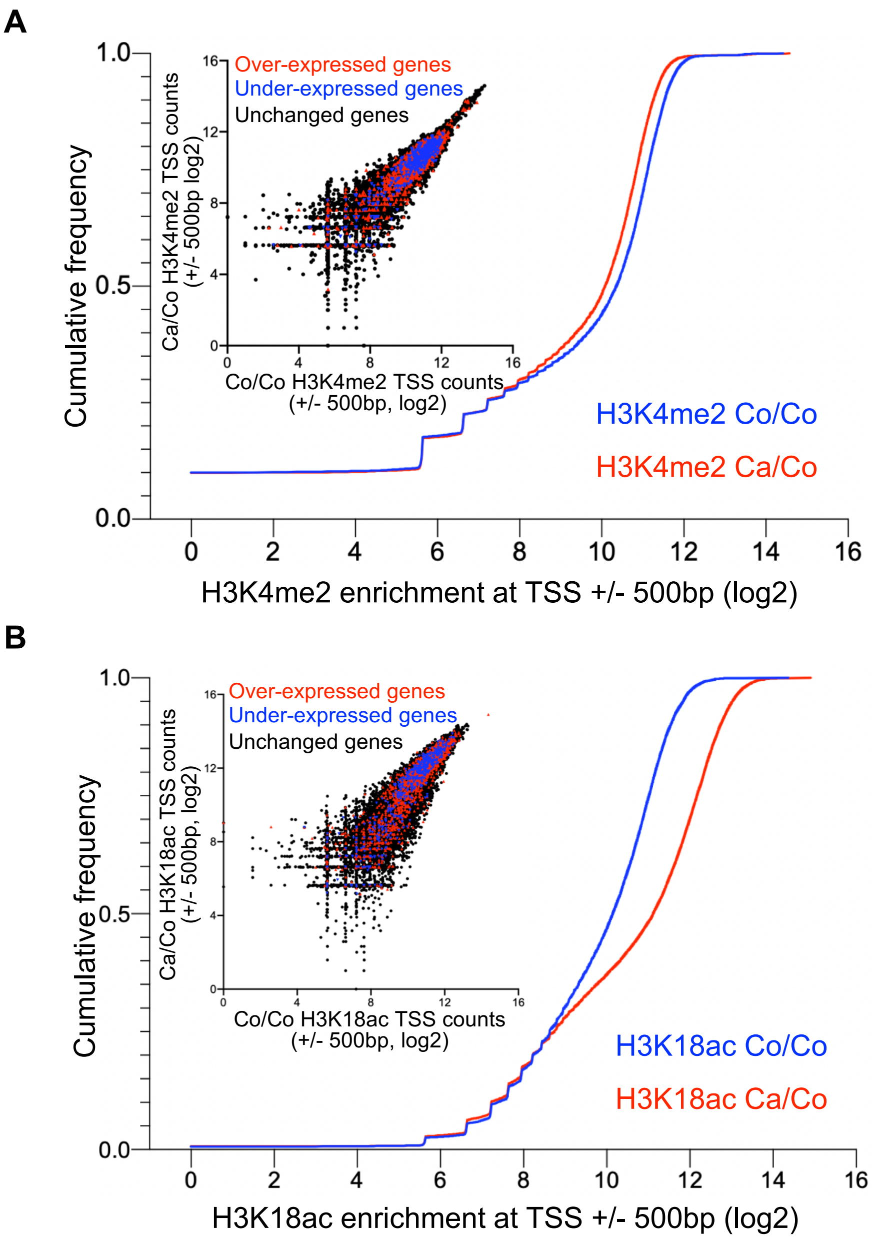

Supplementary Fig 6. A) A cumulative frequency plot showing an decrease in H3K4me2 marks around TSS (+/- 500bp) in Ca/Co group. An inset scatter plot representing the changes in H3K4me2 levels and its correlation of over-expressed (red), under-expressed (blue), and unchanged (black) genes. **B)** A cumulative frequency plot showing an increase in H3K18ac marks around TSS (+/- 500bp) in Ca/Co group. An inset scatter plot representing the changes in H3K18ac levels and its correlation of over-expressed (red), under-expressed (blue), and unchanged (black) genes.

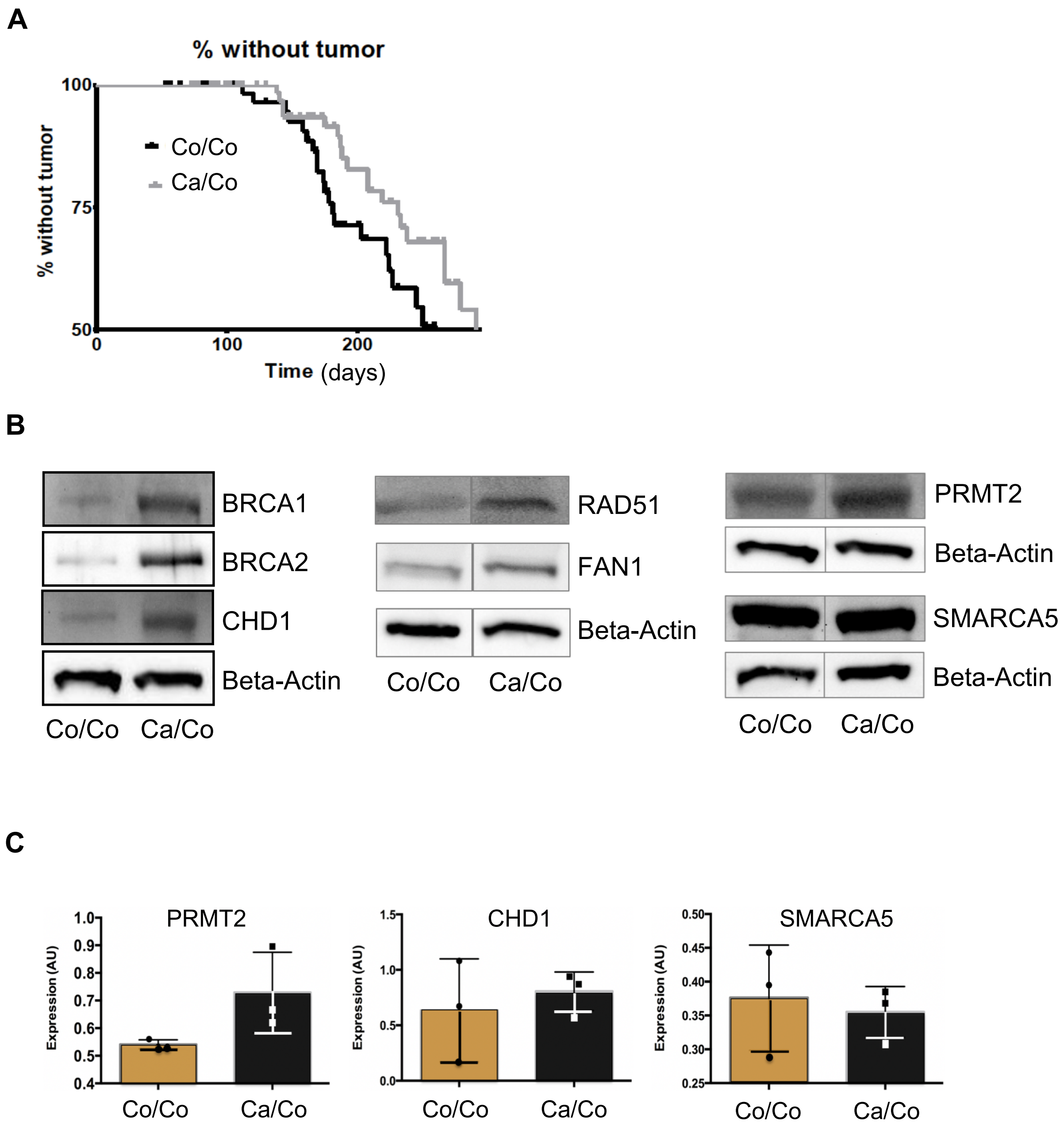

Supplementary Fig 7. Effects of maternal diet on tumor growth and gene expression in DMBA treated mice. A) Percent of mice without tumors after DMBA treatment in F1 mice whose mothers were fed either corn (Co/Co) or canola (Ca/Co) oil rich diets (statistically not significant) **B)** Western Blotting of representative protein expression in mammary tissues. **C)** Graphs representing PRMT2, CHD1, and SMARCA5 proteins expression level quantified using ImageJ after Western Blotting (statistically not significant).

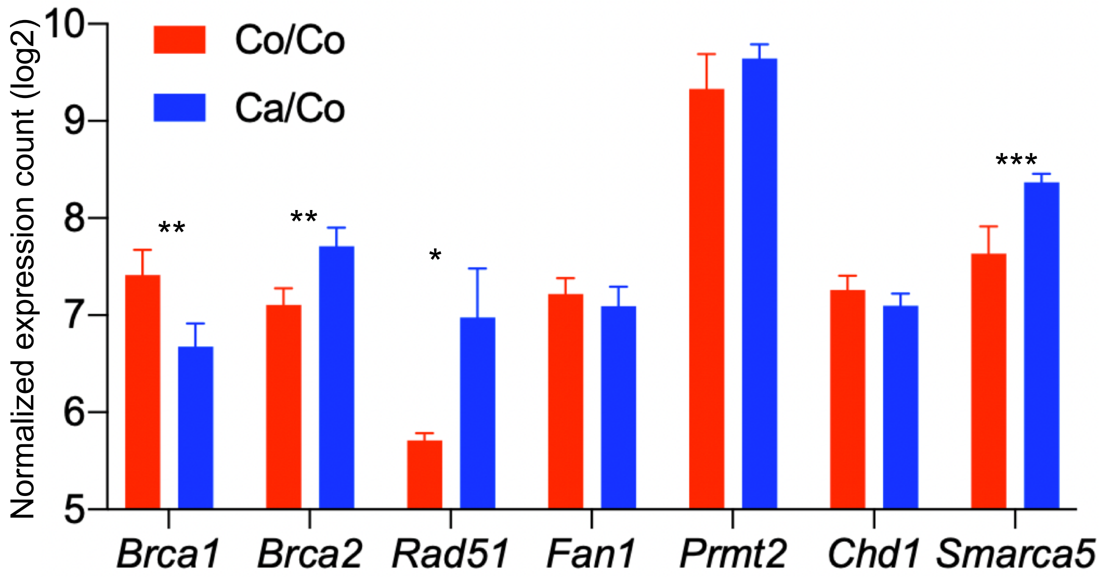

Supplementary Fig 8. Comparison of the transcriptional expression (microarray) of genes in mammary tissue of F1 mice (Co/Co vs. Ca/Co) (* p-value < 0.05, ** p-value < 0.01, and *** p-value < 0.01; mean with SD)
